# Fracture Tables

**Published:** 1849-04

**Authors:** F. H. Hamilton


Buffalo Medical Journal and

Monthly Review.

Vol. 4. APRIL, 1849. No. 11.

### ORIGINAL COMMUNICATIONS.

ART. I.—*Fracture Tables. Showing the Results of Treatment in one hundred and thirty-six Cases.* By Dr. F. H. Hamilton.

The following tables have been made for the purpose of determining the average results of treatment in fractures. The cases, 136 in number, have been collected from various sources, but in no one instance has a case been admitted which I knew to have been managed by an empiric. A few have been furnished by the Buffalo Hospital of the Sisters of Charity, of the surgical wards of which I have the charge; some from my own private practice; twenty or thirty of the ancient fractures were supplied by the medical students in attendance upon the lectures at Geneva and Buffalo Medical Colleges, in reply to my request that such members of the class as had received fractures would call upon me and permit me to make a record of their cases. These young gentlemen being generally from the better classes of society, it was presumed would have received the best treatment which the country could furnish. The remainder have been obtained casually, or as they have been directed to me for the purpose of examination by physicians who have themselves treated the cases, and who wished to aid me in my investigations. No case has been sent to me by one physician which had been treated by another. In short I have sought to make the tables a fair representation of the results of the practice of skillful surgeons, or at least of those who by us are regarded as qualified to practice surgery. If the results are not equal to the expectations of the friends of our science, let them first reflect that no such tables have before been made, and that whatever opinions they may have entertained, such opinions were not based upon general statistics, but only upon individual experience. I wish them also to examine again their own cases, and if they have relied solely upon the statement of the patients, or of their friends, that no shortening exists, they will find, I think, a great many instances in which a careful and proper admeasurement will prove that both were deceived. A shortening of half an inch in the lower extremities will frequently produce a manifest halt, while, on the contrary, I have seen a shortening of an inch which was entirely concealed in the gait. But especially are both patients and friends liable to be deceived in relation to the shortening of a humerus, or even of a clavicle.

If any one shall be so ungenerous as to charge that such may be the results of Erie County surgery, but not of American surgery, I reply, that a very large proportion of the patients have come from other counties and states; some from the hospitals of large cities, and that even foreign hospitals have a respectable representation of bad cases.

The number of fractures is by no means sufficient to warrant positive inferences, but they certainly ought to be regarded as furnishing approximative evidence. We hope that others will occupy themselves in constructing similar tables, by which the fairness of these may be in some manner tested; for we are certainly as desirous as any one that this branch of surgery should not be disparaged.

Some will be surprised that I have always omitted to mention whether the fracture was oblique or transverse. This is an important condition by which to determine the ease or difficulty of adjustment and retention. But in very few of the ancient fractures could this be ascertained; and I am ready to confess that, with regard to the femur at least, when recently broken, I am not often able to determine positively whether the fracture is transverse or oblique. But as in children the fracture is generally transverse, while in adults it is more often oblique, the age, which is given in each case, will enable the reader to form some opinion as to the frequency of the one or the other.

In the tables *y.* is written for *year*, *m.* for *month*, *w.* for *week*, *u.* for *united*, *n. u.* for *not united*, *in.* for *inch*, *p.* for *perfect* and *i. p.* for *imperfect.* I call a limb "perfect" when there is no striking deformity, shortening or maiming.

| | Names of bones. | Point of fracture. | Character of fracture. | Name of patient. | Time since it occurred. | Present age of Patient. | United or not united. | Amount of shortening. | Perfect or imperfect. |
|---|---|---|---|---|---|---|---|---|---|
| 1 | Ossa nasi | | simple | S. L. | 12 *y.* | 22 *y.* | *u.* | | *ip.* |
| 2 | " " | | " | M. L. P. H. | 20 *y.* | 27 *y* | *u.* | | *ip.* |
| 3 | " " | | compound | G. R. | 2 *m.* | 24 *y.* | *u.* | | *ip.* |
| 4 | Vomer | | | W. W. | 8 *y.* | 23 *y* | | | *ip.* |
| 5 | Inf. maxilla | shaft | comp. com. | V. W. | 1 *y.* | 24 *y.* | *u.* | | *p.* |
| 6 | " " | " | " " | N. B. | 8 *y.* | 23 *y* | *u.* | | *p.* |
| 7 | " " | " | " " | J. | 8 *y.* | 25 *y.* | *u.* | | *p.* |
| 8 | " " | " | " " | P. | 2 *y.* | 45 *y* | *n.* | | *ip.* |
| 9 | " " | " | " " | C. B. | 3 *y.* | 55 *y.* | *u.* | | *ip.* |
| 10 | Clavicle | out $\frac{1}{3}$ | simple | K. | 2 *y.* | 4 *y.* | *u.* | | *p.* |
| 11 | " | " | " | Miss M'K. | 2 *y.* | 18 *y.* | *u.* | | *p.* |
| 12 | " | " | " | H. W. | 3 *y.* | 27 *y.* | *u.* | $\frac{1}{4}$ *in.* | *ip.* |
| 13 | " | " | " | A. C. | 5 *w* | 40 *y.* | *u.* | | *p.* |
| 14 | " | " | commin. | B | | 40 *y.* | *u.* | $1\frac{1}{4}$ *in.* | *ip.* |
| 15 | " | " | simple | B. | 1 *y.* | 41 *y.* | *u.* | $1\frac{1}{4}$ *in.* | *ip.* |
| 16 | " | " | " | S.C. | 2 *y.* | 7 *y.* | *u.* | | *p.* |
| 17 | " | mid. | " | Mrs. R. | 34 *y.* | 40 *y.* | *u.* | | *ip.* |
| 18 | " | " | commin. | C. C. | 14 *y.* | 63 *y.* | *u.* | 1 *in.* | *ip.* |
| 19 | " | out. $\frac{1}{3}$ | simple | J. P. | 2 *y.* | 24 *y.* | *u.* | $\frac{1}{2}$ *in.* | *ip.* |
| 20 | " | " | " | W. | 1 *y.* | 46 *y.* | *u.* | $\frac{1}{2}$ *in.* | *ip.* |
| 21 | " | " | " | F. G. | 4 *y.* | 40 *y.* | *u.* | | *p.* |
| 22 | " | " | " | " " | 3 *y.* | 40 *y.* | *u.* | | *ip.* |
| 23 | " | " | " | S. E. | 8 *w.* | 25 *y.* | *u.* | $\frac{1}{2}$ *in.* | *ip.* |
| 24 | " | " | commin. | L. | 9 *w.* | 40 *y.* | *u.* | $\frac{3}{4}$ *in.* | *ip.* |
| 25 | " | mid. | " | | 8 *m.* | 29 *y.* | *u.* | 1 *in.* | *ip.* |
| 26 | " | out. $\frac{1}{3}$ | simple | C. U. H. | 49 *y.* | 68 *y* | *u.* | | *ip.* |
| 27 | " | " | " | P. R. | 3 *m.* | 24 *y* | *u.* | | *p.* |
| 28 | Acromion process, external to junct. of clav. with p. | | simple | S. P. K. | 4 *y.* | 25 *y.* | *u.* | | *ip.* |

2. Left nostril much obstructed—nose turned to right.

4. One nostril is nearly closed, and has had a catarrh ever since.

5. Broken on both sides by a club, through centre of shaft. A small piece exfoliated several months after and came out externally.

6. Broken on both sides, by the heel of a man's boot. United very slowly.

7. Broken on both sides by a brick bat. Had much trouble with it.

10. Treated with Fox's apparatus. It was a *bend* rather than *fracture.*

11. An extraordinary case of a partial fracture with *bending* in an healthy adult.

13 Fragments were never displaced.

14 Broken in three pieces. Central piece stands nearly at right angles with the shaft. He says he has nearly lost the use of the arm from the accident.

15 This is the same case as the above, refractured by being thrown from a carriage.

17 Got out of place three times, slight deformity still remains—has been a little lame at times since the accident.

18 No axillary pad was used; shoulder drops; central fragment turned nearly at right angles with shaft; can use arm as well as ever.

19 Dressed ten days after fracture; used Fox's apparatus; ascribes the deformity to his own carelessness; arm not quite as strong as the other; gaining.

23. Used a cross on back, and axillary pad; was united in fourteen days.

25. The central piece is much out of line: use of arm complete.

28. United by ligament.

| | Names of bones. | Point of fracture. | Character of fracture. | Name of patient. | Time since it occurred. | Present age of patient. | United or not united. | Amount of shortening. | Perfect or imperfect. |
|---|---|---|---|---|---|---|---|---|---|
| 29 | Humerus | mid. 1/3 | " | B. F. McC. | 8 *w.* | 11 *y.* | *u.* | | *p.* |
| 30 | " | up. 1/3 | " | B. | 3 *y.* | 68 *y.* | *u.* | | *p.* |
| 31 | " | low 1/3 | " | J. G, | 10 *w.* | 40 *y.* | *u,* | | *ip.* |
| 32 | " | " | " | B. G. M'K. | 23 *y* | 27 *y.* | *u.* | | *ip.* |
| 33 | " | " | comp. com. | S. H. H. | 17 *y* | 24 *y.* | *u.* | | *p* |
| 34 | " | " | simple | A. G. E. | 15 *y.* | 22 *y.* | *u.* | ½ *in.* | *ip.* |
| 35 | " | " | " | C. C. | 30 *y.* | 63 *y* | *u.* | ½ *in.* | *ip.* |
| 36 | " | ext. c. | " | H. | 3 *m.* | 8 *y.* | *u.* | | *ip.* |
| 37 | " | low 1/3 | comp. com. | H. U. | 8 *m.* | 35 *y* | *u.* | | *ip.* |
| 38 | " | cond. | simple | J. R. S, | 18 *y.* | 21 *y.* | *u.* | ¾ *in.* | *ip.* |
| 39 | " | mid. | " | A. L. | 5 *y.* | 30 *y.* | *n. u.* | | *ip.* |
| 40 | " | low 1/8 | betw'n con. | P. O'B. | 3 *m.* | 10 *y.* | *u.* | | *p.* |
| 41 | " | int. c. | simple | C. C. | 3 *m.* | 11 *y.* | *u.* | | *p.* |
| 42 | " | low 1/8 | " | T. | 4 *y.* | 24 *y* | *u,* | | *ip.* |
| 43 | Radius | neck | " | E. F. | 1 *y* | 12 *y* | *u,* | | *ip.* |
| 44 | " | low 1/3 | " | S. | 6 *y.* | 22 *y.* | *u.* | | *p.* |
| 45 | " | " | " | S. | 1 *y.* | 14 *y.* | *u.* | | *p.* |
| 46 | " | " | " | J. B. | 4 *y.* | 21 *y.* | *u.* | | *p.* |
| 47 | " | mid. | " | P. K. S. | 11 *y.* | 22 *y.* | *u.* | | *p.* |
| 48 | " | low 1/3 | " | S. J. B. | 10 *y.* | 22 *y.* | *u.* | | *p.* |
| 49 | " | " 1/4 | " | Mrs. G. | | 44 *y.* | *u.* | | *ip.* |
| 50 | " | " 1/8 | " | Mrs. C. | 2 *y.* | 40 *y.* | *u.* | | *ip.* |
| 51 | Ulna | olec p. | simple | S. D. | 6*w.* | 14*y.* | *u.* | | *p.* |
| 52 | " | mid. | compound | C. C. | 2*y.* | 11*y.* | *u.* | | *p.* |
| 53 | " | " | simple | H. G. | 3 *y.* | 12*y.* | *u.* | | *p* |

30. This was a fracture through the surgical neck.

31. Elbow stiff—wishes to know whether he ought not to prosecute!

32. Arm much bent at seat of fracture; cannot supine hand completely; ulnar nerve very sensitive; inner half of arm and two small fingers are numb; power of arm much impaired.

33. About one year after accident a piece of bone came out.

34. Slightly bent; not as strong as the other arm and considerably smaller.

36. External condyle is displaced; joint rather stiff.

37. Elbow anchylosed.

38. Condyles separated ½ an inch; much grating between articular surfaces; cannot perfectly straighten the arm; arm occasionally becomes lame; muscles of arm wasted.

42. Bent inwards slightly.

43. Very little power of flexion or extension; no supination.

44. Fracture was oblique; lower end of ulna dislocated at the same time.

46. Both ulnas were slightly displaced at lower end at same time, and still remain so; wrists weak.

49. Ends of fragments pitched in; ulna thrown out; cannot prone hand; many years since the fracture occurred.

50. Lower end of ulna slightly displaced inward; os lunare slightly displaced backward; has had very little use of arm since the accident; it is tender; often painful, and constantly weak.

52. An anterior luxation of the head of the radius at the same time.

| Names of bones. | Point of fracture. | Character of fracture. | Name of patient. | Time since it occurred. | Present age of patient. | United or not united. | Amount of shortening. | Perfect or imperfect. |
|---|---|---|---|---|---|---|---|---|
| 54 " | " | compound | M. A. S. | 3 *y.* | 11*y.* | *u.* | | *p.* |
| 55 " | low $\frac{1}{3}$ | simple | C. H. B. | 2 *y.* | 23*y.* | *u.* | | *p.* |
| 56 " | " | " | Mrs. E. S. | 10*w.* | 26*y.* | *u.* | | *p.* |
| 57 " | " | " | Mrs. —— | 8*w.* | 25*y.* | *u.* | | *p.* |
| 58 " | " | " | C. P. | 4*m* | 38*y.* | *u.* | | *p.* |
| 59 " | up. $\frac{1}{3}$ | " | E. O. B. | 3 *y.* | 35*y.* | *u.* | | *ip.* |
| 60 radius and ulna | low $\frac{1}{3}$ | " | S. D. | 9*w.* | 14*y.* | *u.* | | *ip.* |
| 61 " " | " | " | Mrs. B. B. | 6*w.* | 30*y.* | *u.* | | *p.* |
| 62 " " | " | " | H. A. T. | 21 *y.* | 31*y.* | *u.* | | *p.* |
| 63 " " | | " | G. B. P. | 13 *y.* | 21*y.* | *u.* | | *p.* |
| 64 " " | " $\frac{1}{4}$ | " | L. | 1 *y.* | 10*y.* | *u.* | | *ip.* |
| 65 " " | " | " | H. N. | 10 *y.* | 26*y.* | *u.* | | *p.* |
| 66 " " | " | " | Miss H. | 1 *y.* | 7*y.* | *u.* | | *p.* |
| 67 " " | " | commin. | T. K. S. | 11 *y.* | 50*y.* | *u.* | | *ip.* |
| 68 " " | " | simple | P. M. | 1 *y.* | 7*y.* | *u.* | | *p.* |
| 69 " " | " $\frac{1}{3}$ | compound | L. B. | 2 *y.* | 35*y.* | *n. u.* | | *ip.* |
| 70 " " | up. $\frac{1}{3}$ | simple | N. | 1 *y.* | 9*y* | *u.* | | *ip.* |
| 71 " " | mid. $\frac{1}{2}$ | " | J. L. S. | 3*m.* | 26*y.* | *n. u.* | | *ip.* |
| 72 " " | low $\frac{1}{4}$ | " | J. | 3*m.* | 7*y.* | *u.* | | *p.* |
| 73 rib 4th | | " | F. G. | 5 *y.* | 40*y.* | *u.* | | *p.* |
| 74 rib 10th | an[illegible] $\frac{1}{3}$ | " | B. N. | 6 *y.* | 30*y.* | *u.* | | *ip.* |
| 75 rib 6th | " | " | Mr. M. | 2 *y.* | 45*y.* | *u.* | | *p.* |
| 76 rib 3d | " | " | W. | 1 *y.* | 40*y.* | *u.* | | *ip.* |
| 77 post. sup. spin. p. ileum | | " | Miss R. | 4*m.* | 20*y.* | *u.* | | *ip.* |
| 78 neck of femur | | " | R. | 4 *y.* | 9*y.* | *u.* | $\frac{1}{2}$*in.* | *ip.* |
| 79 " " | | " | Miss B. | 3 *y.* | 53*y.* | *u.* | | *ip.* |
| 80 " " | | " | Mr. E. | 1 *y.* | 78*y.* | *u.* | | *ip.* |
| 81 " " | | " | Mrs. S. | 1 *y.* | 77*y.* | *u.* | | *ip.* |
| 82 " " | within c. | " | J. C. B. | 4 *y.* | 52*y.* | *u.* | 1$\frac{1}{2}$*in.* | *ip.* |

55. Lower end of radius luxated backwards and outward at same time; circumference of wrist $\frac{3}{4}$ of an inch greater than left wrist; cannot supine hand completely.

59. Lower end of ulna is displaced also; at seat of fracture much bent.

60 Much bent—was not treated for a fracture.

61. Was left unreduced two weeks.

62. For three or four years after the fracture a deformity was so apparent that some talked of refracturing it. It is now impossible to discover the seat of fracture.

64. This was two weeks unreduced, at the end of which time I reduced it as perfectly as the callus would permit. A slight bend remains.

65. But the ulna is displaced inward $\frac{1}{4}$ of an inch.

67. The ulna had been broken before and was crooked; and I did not have the case under treatment until the third week after the accident. Deformity is slight.

69. The ulna had united shortened; the radius was not united at the end of two years; power of pronation and supination lost, I sawed off the end of fragments and replaced them; they united and made a good arm.

71. Ulna united, but radius still moveable.

77. Projects.

78. It did not unite under a year; limb is turned in slightly and weak.

81. Shortened; has not used the limb since the accident; is bedridden.

82. See this case and the *trial* reported in the present vol. of this Journal.

| Names of bones. | Point of fracture. | Character of fracture. | Name of patient. | Time since it occurred. | Present age of patient. | United or not united. | Amount of shortening. | Perfect or imperfect. |
|---|---|---|---|---|---|---|---|---|
| 83 " " | | " | Mrs. J. C. | 3 *y.* | 76*y.* | *u.* | | *ip.* |
| 84 " " | | " | Mrs. W. | 7 *y.* | 59*y.* | *u.* | ¾*in.* | *ip.* |
| 85 shaft of femur | mid. ⅓ | " | H. | 3 *y.* | 8*y.* | *u.* | ½*in.* | *ip.* |
| 86 " " | " | " | J. T. | 3*m.* | 14*y.* | *u.* | ¾*in.* | *ip.* |
| 87 " " | low. ⅓ | " | J. B. | 2 *y.* | 30*y.* | *u.* | ½*in.* | *p.* |
| 88 " " | mid ⅓ | " | L. | 8*w.* | 35*y.* | *u.* | ¾*in* | *ip.* |
| 89 " " | " | " | A. M. B. | 2 *y.* | 41*y.* | *u.* | 1 *in.* | *ip.* |
| 90 " " | " | " | J. K. | 8*w.* | 8*y.* | *u.* | | *ip.* |
| 91 " " | " | " | W. S. | 8 *y.* | 22*y.* | *u* | | *p.* |
| 92 " " | " | " | F. | 8*m.* | 5*y.* | *u.* | | *p.* |
| 93 " " | " | " | H. | 2 *y.* | 5*y.* | *u.* | | *ip.* |
| 94 " " | " | " | H. | — | — | *u.* | | *ip.* |
| 95 " " | | | Dr. B. | 2 *y.* | 40*y.* | | 1½*in.* | *ip.* |
| 96 " " | " | " | M. H. | 17*w.* | 25*y.* | | ¾*in.* | *ip.* |
| 97 " " | up ⅓ | " | S. A. | 8 *y.* | 40*y.* | | ½*in.* | *iip* |
| 98 " " | low ⅓ | " | L. | 1 *y.* | 32*y.* | | | *p.* |
| 99 " " | mid. ⅓ | commin. | J. B. | 1 *y.* | 40*y.* | | 1 *in.* | *ip.* |
| 100 " " | " | simple | J. B. | 6 *y.* | 40*y.* | | 1 *in.* | *ip.* |
| 101 " " | mid. | commin. | J. P. | 11 *y.* | 84*y.* | | 2 *in.* | *ip.* |
| 102 " " | | simple | J. P. | — | — | | 2 *in.* | *ip.* |
| 103 " " | | " | W. T. | 5 *y.* | 33*y.* | | 1 *in.* | *ip.* |
| 104 " " | | " | B. L. D. | 20 *y.* | 50*y.* | *u.* | 1½*in.* | *ip.* |
| 105 " " | up. ⅓ | " | J. R. | 3*m.* | 25*y.* | *u.* | | *ip.* |
| 106 top of trocauter major. | | " | M. | 5*w.* | 20*y.* | | | *ip.* |
| | | | | | | | | *ip.* |
| 107 patella. | | " | G. J. | 8*m.* | 5*y.* | | | *id.* |
| 108 " | | " | R. J. | 1 *y.* | 20*y.* | | | |
| 109 Fibula | low ⅓ | simple | L. J. B. | 10 *y.* | 22*y.* | *u.* | | *ip.* |

83. Died before she left her bed.

84. Did not walk in six months; no splints were used.

86. 13 weeks before union occurred.

88. Broken once before at same point, and was then shortened the same.

89. Both legs are much bent, and continue to bend, from a morbid softening.

93 and 94. Both thighs broken at same time. I cannot therefore determine whether any shortening has occurred; they are of the same length, slightly bent.

96. Has a partially anchylosed knee—talks of prosecuting.

101 and 102. Left thigh united shortened two inches; one fracture in right thigh united shortened, and the other did not, but it was shortened four months after the accident two inches; no extension and counter-extension had been used; I made the right leg unite by pressure and rest in a few weeks from this time. Both thighs are now shortened two inches and bent.

103. Surgeon was prosecuted but acquitted.

105. Foot turns out very much; trocauter major very prominent; slipping in joint; often has pain in joint; did not walk for two years after the accident.

106. Fragment is carried upward half an inch.

107. Separated half an inch.

108. This was a very small fragment broken from the upper and inner edge. It remained separated about ¼ of an inch.

109. Displaced inward at seat of fracture; tibia slightly displaced outward; use of limb perfect.

| Names of bones. | Point of fracture | Character of fracture. | Name of patient. | Time since it occurred. | Present age of patient. | United or not united. | Amount of shortening. | Perfect or imperfect. |
|---|---|---|---|---|---|---|---|---|
| 110 " | low $\frac{1}{4}$ | " | E. T. B. | 1 *y.* | 40*y.* | *u.* | | *p.* |
| 111 " | low $\frac{1}{3}$ | " | F. | 2*m.* | 40*y.* | *u.* | | *p.* |
| 112 " | low $\frac{1}{4}$ | " | H. W. | 4*m.* | 38*y.* | *u.* | | *ip.* |
| 113 Tibia | low $\frac{1}{8}$ | compound. | P. M. | 3*m.* | 28*y.* | *u.* | | *ip.* |
| 114 " | up. $\frac{1}{8}$ | simp. tran. | Mrs. W. | 3*m.* | 60*y.* | *u.* | | *p.* |
| 115 Tibia and Fibula | up. $\frac{1}{3}$ | comp. com. | N. K. | 6 *y* | 20*y.* | *u.* | $\frac{1}{2}$*in.* | *ip.* |
| 116 " " | mid. $\frac{1}{3}$ | " " | J. H. | 3 *y* | 38*y.* | *u.* | $\frac{1}{2}$*in.* | *ip.* |
| 117 " " | " | " " | H. C. | 3*m* | 30*y* | *n. u.* | $\frac{1}{4}$*in.* | *ip.* |
| 118 " " | low $\frac{1}{4}$ | " | A. H. | 2 *y.* | 40*y.* | *u.* | | *ip.* |
| 119 " " | low $\frac{1}{3}$ | " | L. D. | 17 *y.* | 31*y* | *u.* | $\frac{1}{2}$*in.* | *ip.* |
| 120 " " | mid. $\frac{1}{3}$ | " " | J S. | 2 *y.* | 40*y.* | *n. u.* | | *ip.* |
| 121 " " | up. $\frac{1}{3}$ | simple | F. G. | 1 *y.* | 40*y.* | *u.* | | *p.* |
| 122 " " | 3 points. | commin. | F. G. | 5 *y.* | 40*y.* | *u.* | $\frac{1}{4}$*in.* | *ip.* |
| 123 " " | low $\frac{1}{3}$ | simple | Mr. H. | 18 *y.* | 68*y.* | *u.* | | *ip.* |
| 124 " " | " | " | A. L. | 2*m.* | 7*y.* | *u.* | | *p.* |
| 125 " " | " $\frac{1}{4}$ | comp. com. | J. C. | 6*m.* | 23*y.* | *u.* | 1 *in.* | *ip.* |
| 126 " " | mid. $\frac{1}{3}$ | " " | J. C, | 6*m.* | 23*y.* | *u.* | 1 *in.* | *ip.* |
| 127 " " | " | " " | M. F. | 1 *y.* | 8*y.* | *n. u.* | | *ip.* |
| 128 " " | low $\frac{1}{3}$ | " | S. | 1 *y* | 9*y.* | *u.* | | *p.* |
| 129 " " | mid. $\frac{1}{3}$ | " " | W. C. | 3 *y.* | 14*y.* | *u.* | 1 *in.* | *ip.* |
| 130 " " | low $\frac{1}{4}$ | simple | R. P. | 8*m.* | 22*y.* | *u.* | | *p.* |
| 131 " " | " | " | Mr. H. | 4*m.* | 35*y.* | *u.* | | *p.* |
| 132 " " | " $\frac{1}{3}$ | comp. com. | W. S. | 7 *y.* | 40*y.* | *u.* | 1 *in.* | *ip.* |
| 133 " " | " | simple | J. H. | 8 *y.* | 48*y.* | *u.* | $\frac{1}{2}$*in.* | *ip.* |
| 134 " " | mid. $\frac{1}{3}$ | compound. | G. | 20 *y.* | 36*y.* | *u.* | $1\frac{1}{2}$*in.* | *ip.* |
| 135 " " | " | simple | A. | 6*m.* | 33*y.* | *u.* | $\frac{1}{2}$*in.* | *ip.* |
| 136 " " | " | compound. | F. M. | 5*y.* | 43*y.* | *u.* | | *ip.* |

112. A dislocation of ankle occurred at same time; patient set it himself; did nothing for fracture; united slightly bent in; use of limb complete.

113. Astragalus broken at same time—ankle partially anchylosed.

115. Produced by fall of a tree on leg; placed on single inclined plane, with foot elevated and moderate extension used; foot sloughed off.

117. Ulceration extended around leg below knee; large slough on heel; also in front; portion of tendon of extensor longus pollicis sloughed out. Prosecuted surgeon.

119. Leg bent slightly at seat of fracture.

120. 13 days after the accident amputation was made; patient died the next day.

122. Broken four inches below knee, at middle and near ankle.

123. For several years he was a little lame; both bones slightly displaced and bent at seat of fracture.

125 and 126. Ant. tib. art. was ruptured.

127. Died in a few weeks.

128. Three months after accident a small piece of bone was exfoliated.

132. Piece of bone sawn off after several months; ankle anchylosed; large irregular deposites of bone; several pieces have exfoliated.

133. There is a lateral inward displacement of tibia of $\frac{1}{4}$ of an inch. Six months before the leg was as good as the other.

136. Necrosis of bone.

SUMMARY.—*Ossa Nasi,* three cases; of which none are perfect; there being more or less deformity in each case.

*Vomer,* one case, with lateral displacement.

*Inferior Maxilla,* whole number five; all in adults; all compound comminuted; all have united. Three are perfect and two imperfect.

*Clavicle,* total eighteen. Perfect six; imperfect twelve. Two of the six perfect cases are children under eight years. Fifteen occurred near the junction of the outer third with the inner two-thirds; three near the middle. Four were comminuted; fourteen simple. I believe not more than three or four were transverse. All have united. Eight shortened from a quarter of an inch to an inch.

*Acromion Process,* one case; united by ligament, but use of arm perfect.

*Humerus,* fourteen. Twelve simple; two compound comminuted; thirteen united, one not united; three shortened from half to three-quarters of an inch. Five perfect; nine imperfect.

*Radius,* eight. All simple. Five perfect; three imperfect.

*Ulna,* nine. Seven simple; two compound. Eight perfect; one imperfect.

*Radius and Ulna,* thirteen. Eleven simple, one compound, and one comminuted. Two not united. Seven perfect; six imperfect.

*Ribs,* four. All simple. Two perfect; two imperfect.

*Posterior Superior Spinous Process of Ileum,* one case, and this left the fragment projecting.

*Femur,* Twenty-eight. Seven through the neck; whether within or without the capsule I have not determined in but one instance. The signs are generally too obscure to make a positive diagnosis. Twenty-six simple; two comminuted. Of twenty broken through the shaft, fourteen are shortened from half an inch to two inches. Only two fractures of the shaft of the femur, out of fifteen cases occurring in adults, are perfect. Probably all the fractures through the neck are shortened, but they were not all measured since recovery.

*Patella,* three. All united by ligament.

*Fibula,* four. Two perfect; two imperfect.

*Tibia,* two. One perfect; one imperfect.

*Tibia and Fibula,* Twenty-two. Seven simple: fifteen compound, or comminuted, or compound and comminuted. Nineteen united: three not united. Twelve shortened from quarter of an inch to an inch and a half. Five perfect; seventeen imperfect.

